# The History of Animal and Plant Sulfite Oxidase—A Personal View

**DOI:** 10.3390/molecules28196998

**Published:** 2023-10-09

**Authors:** Ralf R. Mendel, Günter Schwarz

**Affiliations:** 1 Institute of Plant Biology, Technical University Braunschweig, Humboldtstrasse 1, 38106 Braunschweig, Germany; 2 Institute of Biochemistry, Department of Chemistry & Center for Molecular Medicine, University of Cologne, Zülpicher Strasse 47, 50674 Cologne, Germany; gschwarz@uni-koeln.de

**Keywords:** molybdenum, sulfite oxidase, molybdenum cofactor, sulfur, sulfite oxidase deficiency, molybdenum cofactor deficiency

## Abstract

Sulfite oxidase is one of five molybdenum-containing enzymes known in eukaryotes where it catalyzes the oxidation of sulfite to sulfate. This review covers the history of sulfite oxidase research starting out with the early years of its discovery as a hepatic mitochondrial enzyme in vertebrates, leading to basic biochemical and structural properties that have inspired research for decades. A personal view on sulfite oxidase in plants, that sulfates are assimilated for their de novo synthesis of cysteine, is presented by Ralf Mendel with numerous unexpected findings and unique properties of this single-cofactor sulfite oxidase localized to peroxisomes. Guenter Schwarz connects his research to sulfite oxidase via its deficiency in humans, demonstrating its unique role amongst all molybdenum enzymes in humans. In essence, in both the plant and animal kingdoms, sulfite oxidase represents an important player in redox regulation, signaling and metabolism, thereby connecting sulfur and nitrogen metabolism in multiple ways.

## 1. Introduction

The transition element molybdenum (Mo) is a micronutrient for all kingdoms of life where it forms part of the molybdenum cofactor (Moco) in the active center of enzymes. Computational analyses of molecular evolution showed that Moco was already present in the last universal common ancestor LUCA [1]. While in a number of yeasts, fungi and bacteria, the Mo metabolism was lost during evolution [2], it is essential for all more evolved, multicellular organisms. Only five Mo enzymes are found in eukaryotes (as reviewed by Hille et al. [3]). (1) Sulfite oxidase (SOX), which catalyzes the terminal step in oxidative cysteine catabolism, the oxidation of sulfite to sulfate; (2) xanthine oxidoreductase, which is involved in purine catabolism and reactive oxygen production; (3) aldehyde oxidase, which oxidizes a variety of aldehydes and is essential for the biosynthesis of the stress hormone abscisic acid in plants; (4) nitrate reductase, which catalyzes the crucial step in inorganic nitrogen assimilation in autotrophic organisms; and (5) mitochondrial amidoxime-reducing component (mARC), which is the least studied enzyme and the latest member of the family. A general drug-metabolizing function is assumed, but the physiological substrates are not known for mARC enzymes. In this Special Issue of the journal, the pioneers in the field shed light on the history of how Mo enzymes in all kingdoms of life were discovered and, from a personal perspective, how these researchers studied them in detail. Russ Hille writes on xanthine oxidase [4], Takeshi Nishino writes on xanthine dehydrogenase [5], and José Moura writes on aldehyde oxidoreductase [6], Bernd Clement describes the history of mARC [7], Silke Leimkühler writes on formate dehydrogenase [8], and as the authors of this review, we will present the history of SOX research in mammals and plants.

## 2. The History of SOX

### 2.1. How Everything Began

The occurrence of Mo in living organisms was demonstrated in 1930 when Bortels [9] described Mo as catalytically crucial for biological nitrogen fixation in the bacterium *Azotobacter*. In 1932, Ter Meulen [10] chemically identified Mo in plants and mammals (especially rich in the liver). Two decades later, Mo was identified as a catalytically essential element in purified xanthine oxidase from liver [11] as well as in liver aldehyde oxidase [12] and in fungal nitrate reductase from *Neurospora crassa* [13]. At the same time, the degradation of cysteine in animals was studied in the laboratory of Phil Handler at Duke University, North Carolina. Although it was previously known that the degradation of cysteine by liver preparations proceeds by stepwise oxidation before finally yielding sulfate [14], it remained open whether the last step, i.e., the oxidation of sulfite to sulfate, is an enzymatic step. Irwin Fridovich, a postdoctoral researcher with Handler, took on the project and showed the existence of an enzyme in mammalian liver and kidney being capable of catalyzing this step [15]. In a series of papers, he studied this enzyme in greater detail [16,17]. In 1960, K.V. Rajagopalan joined the laboratory of Phil Handler and started to work on the Mo-enzyme aldehyde oxidase together with Fridovich. He purified the enzyme from liver and characterized it in detail [17,18], also later by using electron spin resonance (EPR) spectroscopy, which was a new and promising technique for analyzing metalloflavoproteins [19]. In the late 1960s, SOX again became a focus of research at Duke University. The importance of this enzyme was emphasized by the discovery of a human child apparently lacking hepatic SOX activity [20]. The child died from severe pathophysiological consequences. With the arrival of Dale Kessler and Harvey Cohen, the fulminant research phase on mammalian SOX started in Rajagopalan’s laboratory. Cohen purified and characterized it from bovine liver [21], followed by Kessler [22] who did the same for SOX from chicken liver.

**Figure 1:**
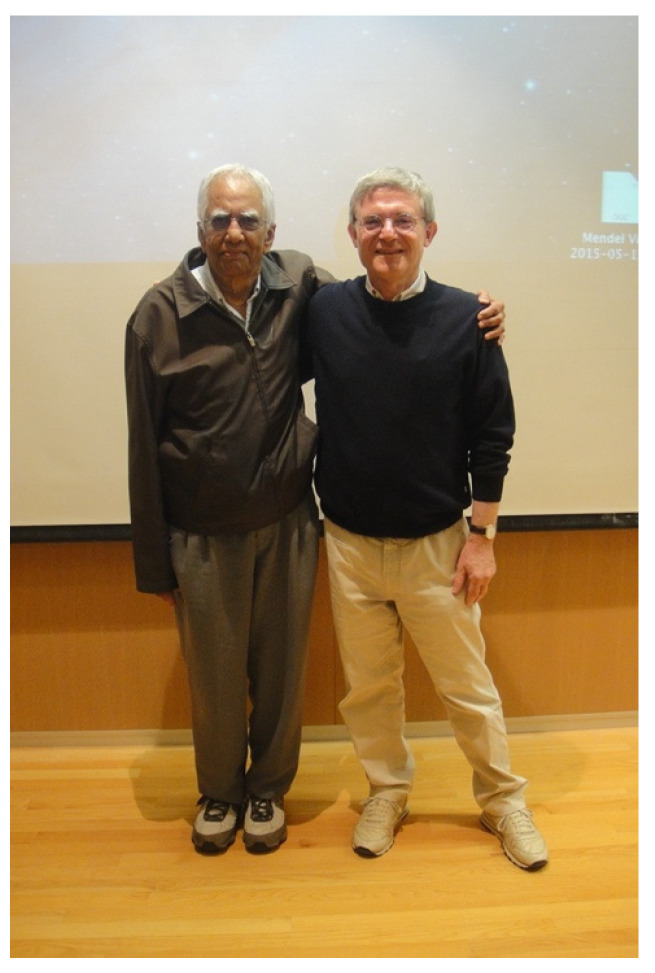
K.V. Rajagopalan and Ralf R. Mendel at Duke University (North Carolina, USA) 2015.

In the 1970s, Jean Johnson arrived at the Rajagopalan laboratory as a predoctoral trainee. While Cohen and Kessler left the group after some time, Jean Johnson stayed with Rajagopalan for several decades. Jean started her very successful research career by focusing on the Mo center of SOX and creating Mo-free SOX from the livers of rats that had incorporated tungsten [23], and was characterized in all detail including EPR spectroscopy (tungsten is an antagonist of Mo and renders Mo-enzymes inactive). Shortly after, she analyzed the frozen autopsy liver tissue of a child that had died presumably due to SOX deficiency [20]. She found that the SOX protein was not detectable using antibodies directed against purified rat liver SOX, while the activity of another Mo-dependent enzyme, xanthine oxidase, was normal [24]. Consistently, the level of Moco was normal in this patient as quantified by an in vitro-reconstitution assay that had been set up in Rajagopalan’s laboratory (purified rat SOX-apoprotein free of Moco could be reconstituted by adding enzyme preparations from other sources [25]). At that time, Rajagopalan started a collaboration with the group of Vivian Shih at the Harvard Medical School which identified another SOX-deficient patient. The boy had strong neurological aberrations accompanied by large amounts of abnormal sulfur metabolites including sulfite in the urine [26], later it was again Jean Johnson clarifying that this patient was Moco-deficient laying the foundation that, clinically, the SOX- and Moco-deficiency are very similar in underlying the prime role of SOX amongst all Moco-dependent enzymes in vertebrates (see Section 4 of this review). This work triggered Jean Johnson’s lifelong interest in studying human SOX and Moco deficiency from a biochemical perspective, which we will discuss below in the paragraph “SOX deficiency”. In parallel, Jean focused on two other research lines, namely to identify the chemical nature of Moco and to characterize SOX in ever greater detail. The history of Moco research was recently described [27] and will not be further discussed here. Before we continue to shed light on the biochemistry of the SOX enzyme in the next section, we have to add an interesting note. In 1969, Phil Handler left Duke University to become the President of the National Academy of Sciences of the USA (a position that he held for 12 years) and he designated Rajagopalan as the PI of his National Institutes of Health (NIH) grant. This subsequently became the longest continuously funded NIH grant in the U.S.A. [28]. Figure 1 shows K. V. Rajagopalan together with Ralf Mendel in 2015. 

### 2.2. Early Biochemistry of SOX: Cows, Dogs, Chicken, and Rats (the 1950s, 1960s, and 1970s)

As mentioned above, Irwin Fridovich started to study mammalian SOX in the 1950s. Partial purifications of rat liver resulted in a fraction that gave initial enzymatic data [15], and this work was continued with partial purifications from dog liver (e.g., inhibition with sulfhydryl reagents) [16]. A milestone was marked by the 1961 paper [29] that presented the purification schemes for SOX from rat liver, dog liver, and beef liver (ranging from 200–300-fold) with the following key parameters:

- pH optimum 8.5;
- Km 2 × 10^−5^ M (sulfite);
- Km 3–9 × 10^−7^ M (cytochrome *c*);
- cyt *c*, oxygen, ferricyanide were reduced by the enzyme;
- p-chloromercuribenzoate inhibited the enzyme;
- absorption spectrum typical for cyt *b*_5_;
- formation of peroxide during catalysis;
- most activity found in the microsomal fraction;
- high amounts in liver, kidney, heart (rat);
- relatively low amounts in brain, lung and spleen (rat);
- no flavin found.

A decade later, Irwin Fridovich trained Harvey Cohen as a Ph.D. student who purified SOX from bovine liver 1000-fold in order to refine the data published earlier by this group [21]. In the accompanying paper of Cohen (together with Fridovich and Rajagopalan) [30], the authors supposed that there must be another chemical group—in addition to heme—that is involved in electron transfer from sulfite to oxygen, and they identified Mo as a component of this functional second group. This paper made extensive use of EPR spectroscopy, a technique that Rajagopalan had previously used for aldehyde oxidase [19]. The oxidized enzyme gave no detectable EPR signal at the temperature of liquid nitrogen. Upon reduction, a strong signal did appear “whose similarity to previously described Mo signals left little doubt as to its significance” [30]. Chemical analysis confirmed the presence of Mo in equimolar amounts with heme. No flavin was found. 

The Suzuki laboratory had previously published the results of isolating and characterizing SOX from the bacterium *Thiobacillus* [31] with properties strongly differing from the mammalian enzyme. In view of this controversy, Rajagopalan decided to characterize SOX from a species of intermediate phylogenetic level, and he chose chicken. At that time, Dale Kessler joined Rajagopalan’s group as a Ph.D. student and performed these experiments [22]. The chicken enzyme was found to be very similar in nearly all respects to the previously purified bovine enzyme. It is noteworthy that Rajagopalan let Dale Kessler also make partial SOX purifications from human liver, *Thiobacillus thioparus*, and wheat germ. All showed similar EPR spectra; however, for the bacterial and plant enzyme, there were no indications for the presence of a heme group [22]. Furthermore, Harvey Cohen identified rat liver SOX in the intermembrane space of mitochondria [32]. 

The metabolic essentiality of SOX was shown in the following way. Indications came in 1971 from Yokoyama et al. [33] who showed that dogs exposed to ^35^SO_2_ gas excreted up to 90% of the sulfur as sulfate in the urine. When Cohen et al. [34] took tungsten-treated rats [23] that were found to become deficient in SOX-activity and subjected them to bisulfite injections, they found that these rats were more susceptible to bisulfite toxicity compared to control rats. They also stated that SOX activity was not inducible by bisulfite. 

Rat liver SOX is a homodimeric enzyme. In 1977, Jean Johnson showed that trypsin-treated rat liver SOX fell into two fragments, the smaller one (M_r_ 9500 Da) harboring the heme domain and the larger one (M_r_ 47,400 Da) retaining the Mo center with all EPR properties seen in the native enzyme [35]. This paper also noted that the Mo center is a weak chromophore with an absorption spectrum suggestive of coordination with sulfur ligands. It was concluded “that the two cofactor moieties of SOX are contained in different domains which are covalently held in contiguity by means of an exposed hinge region” [35]. Johnson and Rajagopalan speculated that a flexible connection between the Mo and heme domain could facilitate electron transfer by moving the heme domain (once reduced by the Mo domain) away from the Mo center to facilitate readier access to the mitochondrial inner membrane components. This paper also showed first crystals of both domains when crystallized separately. William Southerland was another Ph.D. student that joined the Rajagopalan laboratory and characterized the two domains in greater detail, suggesting that the hinge segment is at least 30 residues in length [36]. 

**Figure 2:**
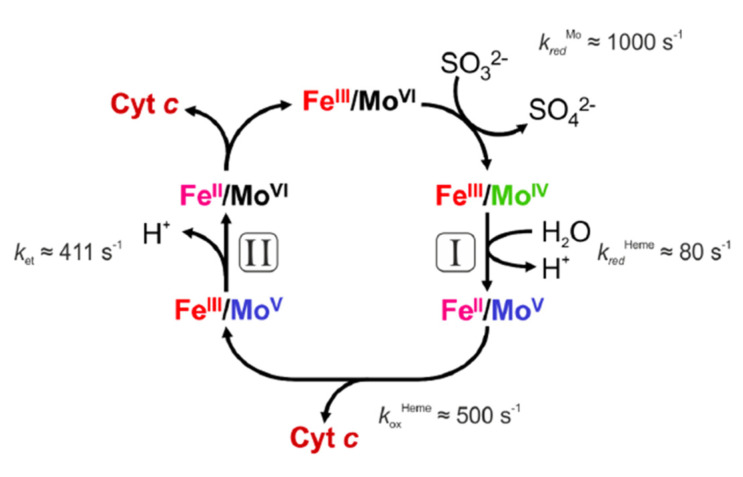
Reaction cycle of vertebrate SOX. In the resting state, Mo^VI^ in Moco will rapidly oxidize sulfite into sulfate, leaving a reduced Mo^IV^ species behind. Rapid kinetics studies demonstrated a rate for the transfer of the first electron from Mo^IV^ to the heme iron of 80 s^−1^ (I), which suggest a slower rate for electron transfer than for catalysis (*k*_red_ = 1000 s^−1^). The second electron is assumed to be transferred with a higher rate (II), as determined by the laser flash photolysis in the Enemark lab.

The published drawing of the proposed domain structure of dimeric SOX was very close to the crystal structure determined two decades later by Caroline Kisker and Hermann Schindelin, two German biochemists that joined the laboratory of Douglas Rees at Caltech Pasadena (USA). The Rees lab was at that time the “center of the universe” in determining crystal structures of Mo- and W-containing enzymes. It is remarkable that the proposed remote position of the heme domains perfectly matched the structural findings determined later. Most importantly, the lack of function, following trypsin treatment, already demonstrated in the early days of SOX enzyme research how important the mobility of the heme domain is to ensure closing the catalytic cycle by connecting electron flow from Mo to cyt *c*, thus contributing to mitochondrial respiration. Figure 2 shows the reaction cycle proposed for vertebrate SOX.

### 2.3. Amino Acid Sequences, Gene Cloning, and First Structure (the 1980s and 1990s)

A decade after the domain structure became clear, Neame and Barber (1989) [37] subjected purified chicken liver SOX peptides to Edman degradation and published the first complete amino acid sequence of avian SOX, comprising 460 amino acids with a M_r_ of 50,545. Surprisingly, the comparison of this sequence with the cDNA-deduced sequence of assimilatory nitrate reductase from the model plant *Arabidopsis thaliana* showed a substantial degree of sequence conservation in the Mo-domain and the heme domain, indicating a common ancestral origin. In an accompanying paper, the authors analyzed the first partial amino acid sequence of rat liver SOX and compared it to the sequence of the chicken enzyme, showing that two cysteines are highly conserved [38]. One of these cysteines is also conserved in plant nitrate reductase which coincides with earlier observations of EXAFS studies that indicated a structural similarity of the Mo active sites of SOX and nitrate reductase [39,40]. This cysteine might be the protein-provided cysteine liganding Mo of the Moco. 

A few years later, Robert Garrett joined the group of Rajagopalan and introduced molecular techniques to the laboratory. He cloned the SOX gene from a cDNA library of rat liver, analyzed it, expressed the protein recombinantly in *E. coli* and purified the encoded protein for further analysis [41]. A 22-residue sequence at the N-terminus was interpreted as the mitochondrial targeting sequence. The recombinant protein harbored the molybdopterin (MPT)-based Moco and not the bacterial dinucleotide Moco-version, demonstrating the SOX was able to selectively extract a eukaryotic form of Moco present in *E. coli*, which at that time was not anticipated given the well-known dinucleotide-modified bis-Moco in bacteria. 

Meanwhile, sequences for nitrate reductase from plants (Arabidopsis and tobacco) and fungi (*Neurospora crassa* and *Aspergillus nidulans*) were available for comparison and showed a considerable degree of similarity (37% identical at the amino acid level). A year later, the gene of human liver SOX was cloned and characterized in Rajagopalan’s laboratory [42]. A single cysteine residue (Cys207 in rat SOX) was invariant in all SOX and nitrate reductase sequences, obviously functioning as a ligand to Mo. To prove this assumption, Garrett performed site-directed mutagenesis on the codon for this cysteine of rat and human SOX followed by spectroscopic analyses [43]. Once the recombinant molecular tools were established in the laboratory, the door was open to a molecular analysis of human patients suffering from SOX deficiency. A young girl with medical symptoms and biochemical characteristics typical for SOX deficiency was analyzed and a R160Q substitution was identified. The variant protein was recombinantly expressed, purified, and analyzed [44]. The content of Mo was normal, yet the enzymatic activity was below 5 per cent. For the first time, kinetic and spectroscopic analyses of SOX with a functionally essential residue were possible and permitted a deeper insight into the enzymatic mechanism of this enzyme. In parallel, the atomic structure of crystallized chicken liver SOX was solved [45], showing that the R160 residue is localized in the Mo active site. 

### 2.4. The Beauty of the SOX Structure and Unanswered Questions

In parallel to uncovering SOX genotype–phenotype correlations, the era of providing three-dimensional structures of Mo- and W-containing enzymes started in 1995. First, the Rees laboratory reported in March the structure on the ferredoxin-dependent W-containing aldehyde oxidoreductase from the thermophile *Pyrococcus furiosus* [46], which was followed a few months later by the first structure of a Mo-dependent aldehyde oxidoreductase from *Desulfovibrio gigas*, determined by Maria Romao while she was a visiting postdoctoral researcher in the laboratory of the Nobel Laureate Robert Huber in Munich (Germany) [47], both of which were published in Science with more than 500 citations each. Although these enzymes were of archaeal or bacterial origin and harbored a special form of Moco, the discovery of a third pyran ring in the pterin cofactor was completely unexpected and resulted in a revision of the chemical structure of pterin-based cofactors. 

**Figure 3:**
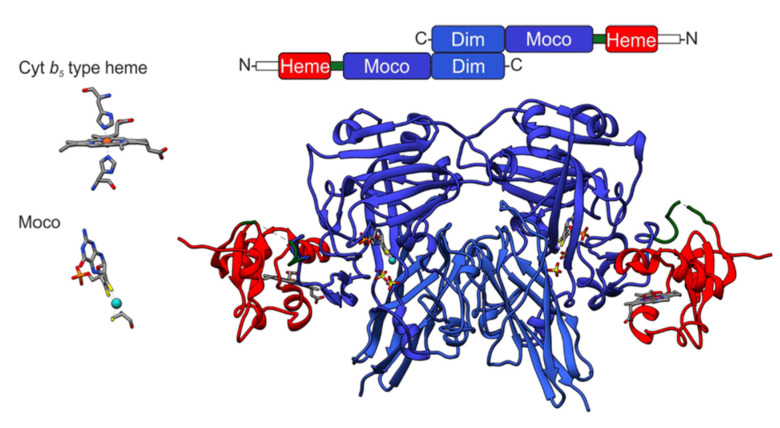
Structure of chicken SOX [45]: the arrangement of the dimeric SOX along the primary sequence, with the N-terminal domain depicted in red, the Moco domain depicted in dark blue, and the dimerization (dim) domain depicted in light blue. Both prosthetic groups including the protein-derived coordinating residues are shown on the side.

Shortly after, a first conference on Mo- and W-containing enzymes was organized in 1997 in Brighton, Sussex (UK), by Robert C. Bray, a pioneer in metal enzyme spectroscopy. Caroline Kisker gave a talk on her recently solved and not yet published crystal structure of SOX, the first of a eukaroytic Mo enzyme, which again showed a tricyclic pyranopterin structure of Moco. The success in the project was a truly collaborative approach given that the enzyme preparation was provided by John Enemark from Tucson, Arizona (USA). His group had prepared the enzyme from more than 20 kg of chicken liver and handed it over to Caroline Kisker, a postdoctoral researcher that had recently joined the Rees laboratory (Figure 3). Besides a novel fold, most surprising was a very remote position of the N-terminal heme domain with a Mo-to-Fe distance of more than 30 Å raising the question how efficient intra-molecular electron transfer may occur [45]. In addition, the previously reported critical Arg160 residue was found to localize within the active site. In addition, numerous other pathogenic variants were mapped onto the structure, providing a first structure-based understanding of disease-causing mutations.

In the following years, the Enemark laboratory dedicated a lot of effort to understand the paradox of a huge Mo–Fe distance observed in the crystal structure of chicken SOX and rapid intra-molecular electron transfer detected by pre-steady state kinetics [48] with laser flash photolysis [49]. Without a doubt, the huge mobility of the heme domain was found to be essential to serve two electron donor (Mo domain) and acceptor (cyt *c*) sites within the dimeric enzyme. Both increasing solution viscosity as well as the shortening of the solvent exposed the highly mobile tether connecting the heme to the Mo domain, negatively impacting the intra-molecular electron transfer rates with a moderate impact on steady state kinetic parameters, suggesting that sulfite oxidation at the Mo center became the rate-limiting step in the reaction.

Another enigma in Mo enzyme research was the role of dimerization in SOX and other enzymes of this family. It has been demonstrated by multiple studies that mutations either found in patients suffering from SOX deficiency [50] or introduced by structure-based mutagenesis [51] abolish catalytic activity. Given that dimerization has been found to be closely linked to Moco insertion [52], it was technically difficult to experimentally dissect those different processes. 

A recent study by the Schwarz laboratory made use of the expression of human SOX modified with two different peptides (6xHis- and myc-tag) that allowed for the purification and biochemical characterization of heterodimeric SOX variants [53]. By using variants with only one heme domain and/or a dysfunctional active site, we were able to determine the electron transfer rate from Mo to the heme within a subunit, between subunits and between individual SOX dimers, unequivocally demonstrating that one half of the SOX dimer is fully functional and does not require the other side of the dimer. Nonetheless, unpublished CryoEM work with Elmar Behrmann (Cologne, Germany) identified different localizations and mobilities in the heme domains for a given subunit within the dimer, suggesting a half-site reactivity of the enzyme, a phenomenon that has been proposed for many dimeric multidomain enzymes [54] but seldom shown or experimentally proven. In aggregate, the dimeric SOX is a highly dynamic and structurally mobile enzyme, which also impacts another moonlighting function, the reduction in nitrite to nitric oxide (see below).

### 2.5. The Multiple Roles of SOX in Metabolism

SOX catalyzes the terminal step in the catabolism of cysteine in animals. Cysteine represents one of the most tightly regulated amino acids in metabolism and only low amounts of free cysteine are found in the cytosol (10–20 µM), while its major form for inter-organ distribution is oxidized cystine present at 200 µM in plasma. Besides proteins, the largest ‘sink’ for cysteine is glutathione, the major antioxidant of the cell reaching up to 25 mM cellular concentrations. Although cysteine in animals is synthesized by the reverse transsulfuration pathway from homocysteine [55], it is considered a semi-essential amino acid due to its precursor methionine. Plants can directly synthesize cysteine from sulfate [56].

The catabolism of cysteine involves various routes with the oxidative pathway harboring the largest load of S-flux towards sulfate as an end product [55]. First, cysteine dioxygenase—a tightly regulated enzyme—initiates the oxidation of the cystine thiolate, forming cysteine sulfinic acid. Recently, we could show that cytosolic aspartate aminotransferase (also named glutamate oxaloacetate transaminase) is the main enzyme converting cysteine sulfinic acid into pyruvate and sulfite [57]. In addition, the synthesis and catabolism of hydrogen sulfide (H_2_S) has gained growing attention in the field. As a result, H_2_S exclusively contributes to the formation of thiosulfate and partially to sulfate as terminal products of cysteine catabolism. Similar to SOX deficiency (see below), defects in H_2_S catabolism are also accompanied by neurodegeneration and developmental delay and/or arrest. 

SOX is localized to the mitochondrial intermembrane space where it transfers electrons to the cyt *c*, thus contributing to respiration. Again, the first enzyme in H_2_S catabolism also feeds electrons into the respiratory chain via the quinone pool. Therefore, it is not surprising that defects or changes in cysteine metabolism come with mitochondrial disorders [58,59].

As a mitochondrial intermembrane enzyme, SOX follows a unique maturation which involves an N-terminal targeting sequence that is cleaved upon transport through the outer mitochondrial membrane and a folding trap mechanism that strictly requires the presence of Moco in order to withhold the enzyme in the intermembrane space of mitochondria [52]. As a result, in fibroblasts derived from patients with defects in the synthesis of Moco causing a loss of function in all Moco enzymes including SOX, the apo-protein of SOX was fully absent (degraded) due to its defect in mitochondrial maturation in the absence of Moco. Interestingly, a new form of SOX deficiency has recently been reported by our group [60,61] that affects the kinetics of Moco binding to SOX apo-protein, thus causing an impaired maturation of the enzyme in eukaryotic cells. 

Following a meeting at Duquesne University in Pittsburgh, Marc Gladwin raised the question of the extent to which SOX has ever been probed for a non-canonical function in nitrite reduction, as had previously been reported for xanthine oxidase [62]. This meeting was the start of Günter Schwarz’ productive collaboration with the Gladwin lab finally showing stoichiometric sulfite- and nitrite-dependent nitric oxide synthesis by the SOX Mo domain [63]. A few years later, two graduate students in his group, Daniel Bender and Alex Kaczmarek, managed to further clarify the reaction mechanism of SOX with nitrite and closed the catalytic cycle in the presence of cyt *c*. Following a sulfite-dependent reduction in the Mo center, the nitrite will bind to Mo^IV^ and will be reduced in a one-electron reduction step to nitric oxide, leaving a Mo^V^ species behind that requires another single electron transfer step to regenerate the Mo^VI^ species for a new catalytic cycle of sulfite oxidation. Most critical in this reaction is the life-time of the fully reduced Mo^IV^ species which will rapidly transfer an electron to the heme. However, conditions that slow this reaction down (such as limiting concentrations of oxidized cyt *c*) will favor nitrite-dependent nitric oxide synthesis by SOX. Current and ongoing research in my laboratory explores the physiological importance of SOX-dependent nitric oxide synthesis with a particular emphasis on mitochondrial function. 

## 3. Plant SOX Is Different—A Personal View by Ralf Mendel (Years 2001 Onward)

In 1974, as a Ph.D. student, I started to work with the two plant Mo-enzymes, nitrate reductase and xanthine dehydrogenase [64,65], but soon shifting to study Moco biosynthesis, and I continued to do this for almost five decades which I recently summarized as a personal review about the history of Moco [27]. After joining the Technical University of Braunschweig as a full professor in 1992, my interests broadened and nitrate reductase [66] and xanthine dehydrogenase [67] returned into the focus of my research in my newly established laboratory. Only plant SOX was missing. This enzyme remained a mystery for 10 more years. Similar to animal experiments described above wherein dogs were exposed to ^35^SO_2_ gas and excreted the label as sulfate [33], plant researchers also exposed plants to ^35^SO_2_ gas and found the label in sulfate [68], underlining the earlier findings of Thomas et al. (1944) [69] who reported sulfite oxidation by SO_2_-treated alfalfa. These experiments showed that, after entering the plant leaf, SO_2_ gas is converted into sulfite once it is dissolved in water. Plants are sulfur autotrophs, i.e., they have to convert inorganic oxidized sulfur (sulfate) via sulfite into organic reduced sulfur (sulfide) that is essential for cysteine biosynthesis [70]. This sulfur assimilation proceeds in the chloroplasts. SO_2_-derived sulfite either follows the reductive pathway [39] to form cysteine or it becomes oxidized to sulfate. But the enzyme catalyzing the latter step was elusive. At the end of the 1990s, there was controversy among the international plant sulfur community. One group was convinced that the putative existence of a plant SOX would contradict sulfur assimilation—why should sulfite as an intermediate metabolite of the sulfate reduction pathway be reoxidized—what a waste of ATP! The other group of researchers was convinced that there must be an enzyme responsible for the observed ^35^SO_2_ conversion to sulfate, and peroxidases were suggested to be involved [71]. 

**Figure 4:**
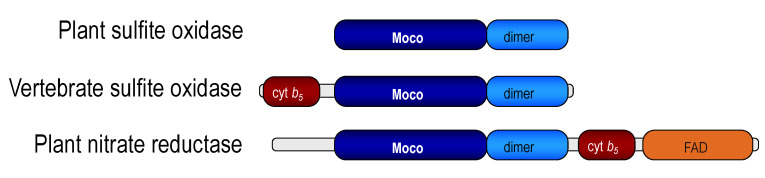
Domain structures of SOX from plant and chicken, and of plant nitrate reductase.

I decided to try to clone the gene of the putative plant SOX on the basis of the sequence data available for animal SOX. One should keep in mind that the plant (*Arabidopsis thaliana*) genome was not yet published at that time, and so Henner Brinkmann, a senior postdoctoral geneticist in my lab, had to use the standard and a little tedious method of cDNA cloning via expressed sequence tags and screening an Arabidopsis cDNA library. To our surprise, the encoded protein was only 393 amino acids residues in size (43.3 kDa) with 47% identity to the Moco domain of chicken SOX, and it was lacking the heme domain known from animal SOX [72]. To exclude the possibility that the identified cDNA was truncated, the genomic structure of this gene was analyzed from a Genbank BAC-clone, but no sequence coding for a heme domain was found upstream or downstream of this gene. Therefore, we had to believe that the Arabidopsis SOX (At-SOX) was different from the animal counterpart. At-SOX showed higher homologies to vertebrate SOX than to the Arabidopsis nitrate reductase (NR) Moco-domain (Figure 4).

**Figure 5:**
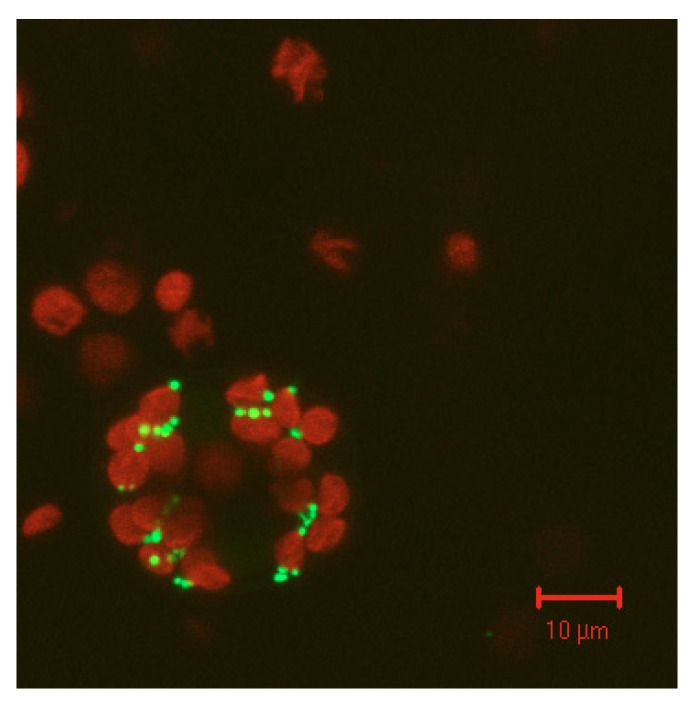
Arabidopsis SOX labelled with GFP is expressed in tobacco leaf cells. When exciting the cells with blue light, green fluorescence is emitted in peroxisomes while chloroplasts (=red large spheres) exhibit red autofluorescence.

After our Ph.D. student Thomas Eilers expressed recombinant At-SOX in *E. coli* and purified the protein, no activity was found with cyt *c* as the electron acceptor. This was expected since the heme domain is known to mediate the electron transfer between the Moco domain and cyt *c* in animal SOX; however, the enzyme was active in the ferricyanide assay in the same range as for rat SOX [72]. At-SOX contained the well-known pterin-type Moco. EPR-spectra were nearly identical to those of the chicken enzyme. Using antibodies raised against recombinant At-SOX, a single band at 45 kDa was detected in the immunoblots of crude extracts of Arabidopsis leaves. A similar single band was obtained when analyzing the crude extracts of leaves from tobacco, pea, barley and poplar trees [72] showing that SOX is widely distributed among herbaceous and woody plants. Subcellular fractionation experiments indicated a peroxisomal localization for plant SOX. Indeed, in silico-analysis revealed that the protein contains a C- terminal SNL tripeptide which is the consensus peroxisomal targeting signal 1 (PTS1) and is sufficient to direct polypeptides to peroxisomes in vivo in plants, animals, and yeast [73]. Therefore, we fused the reporter GFP (green fluorescent protein) to the N-terminus of At-SOX and expressed it transiently in plant leaves where the fluorescence was exclusively found in peroxisomes [74] (Figure 5). When we deleted the PTS1 targeting signal or substituted residues in it, the fluorescence was not seen in peroxisomes but stayed in the cytosol. 

Following the biochemical characterization of At-SOX, a new Ph.D. student Nils Schrader started to crystallize At-SOX in the laboratory of Caroline Kisker at the State University of New York, Stony Brook (USA). Caroline’s lab was the best place to join given her established expertise with chicken SOX and her subsequent work on xanthine dehydrogenase [75]. 

At-SOX was well-expressed in *E. coli*, and the structure was solved at 2.6 Å resolution [76]. The overall fold and the coordination of Moco are similar to the chicken SOX, whilst the only smaller differences were seen in the arrangement of the monomers within the SOX dimers. Comparisons between conserved surface residues and charge distributions in plant SOX and chicken SOX revealed differences near the entrance to both active sites suggesting different exit routes for electrons following sulfite oxidation, which is in line with the different electron acceptors used by vertebrate (cyt *c*) and plant SOX (oxygen). Interestingly, Arg374 was identified as an important residue for substrate binding due to its conformational change in the plant SOX apo-structure compared to the sulfate bound structure of chicken SOX. 

**Figure 6:**
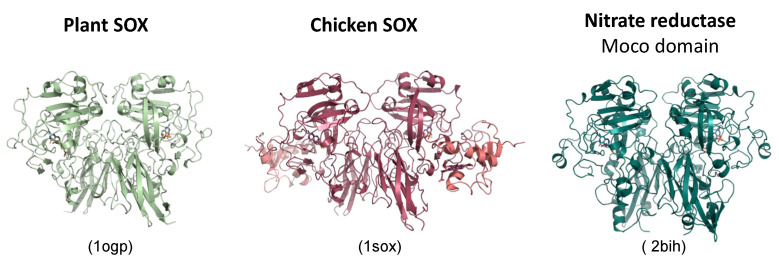
The structural comparison of plant and chicken SOX with the yeast NR Moco domain. Note that the heme and FAD domains of NR are missing. All structures are shown in a comparable orientation highlighting the structural similarity and similar fold. Figures were generated with Pymol using the PDB coordinates shown in brackets.

Given the significant sequence conservation between SOX and plant nitrate reductases (NR), another student, Katrin Fischer, which was also trained in the Kisker laboratory during her Diploma thesis project, decided to dedicate her thesis project to revealing the crystal structure of a plant-type NR. In collaboration with Bill Campbell (Michigan Technological University), we prepared the NR Moco-domain from the yeast *Pichia pastoris*, and following a complex journey of thousands of crystallization trials, Katrin succeeded in collecting a dataset from a single crystal that grew out of precipitated protein within the original screening well. The structure was finally determined at a 1.6 Å resolution and confirmed a remarkable structural similarity with SOX enzymes (Figure 6), except a few but highly significant differences at residues surrounding the active site of the enzyme, enabling us to propose a structure-based reaction mechanism [66].

Among eukaryotes, plant SOX was the smallest molybdenum enzyme known at that time (it later turned out that mARC, as we described in 2006 [77], was even smaller), and it was and is the only one lacking other redox-active centers which made it attractive for spectroscopy. It was a pleasure for us to provide recombinant At-SOX for in-depth spectroscopic analyses to the laboratories of Russ Hille (Ohio State University & UC Riverside, USA) and John Enemark (University of Arizona, USA) to perform EPR and resonance Raman [78] as well as pulsed EPR spectroscopy, respectively [79]. Russ Hille was very surprised when he saw that our plant SOX was extremely fast in catalysis, and he wrote to us “An extensive rapid kinetics study on the reaction of SOX with sulfite was prohibited by the extremely rapid rate of the reaction. Even at 5 °C, substrate oxidation was complete within the dead-time of the stopped-flow apparatus, indicating that *k*red for the plant enzyme is at least 10 times greater than that for the enzyme from chicken liver” [78]. These were only initial experiments, and later, Russ Hille published detailed studies of this very fast enzyme [80]. In light of the conservation/invariance of the active site structure of plant SOX, it was not surprising that the catalytic cycle was found to be similar to the chicken enzyme.

**Figure 7:**
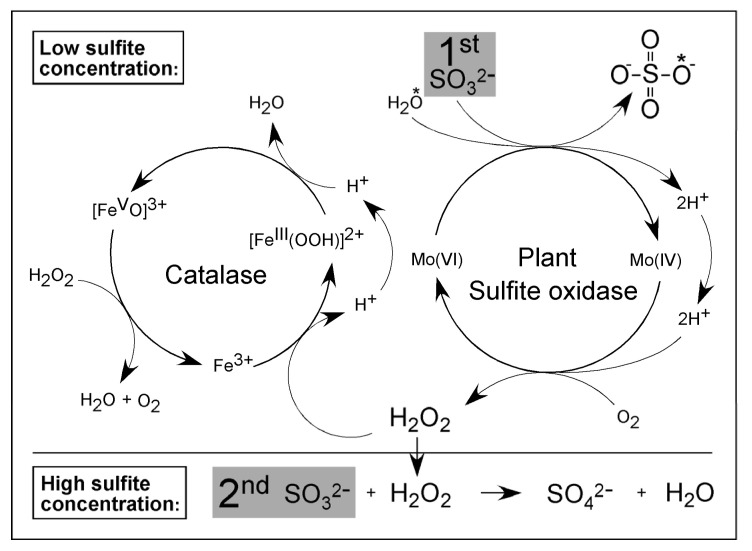
Model of interaction between plant SOX and catalase at higher sulfite concentrations. The asterisk (*) denotes the oxygen atom that is introduced into sulfite to form sulfate. Further explanations are given in the text. Modified after [81].

Plant SOX does not react with cyt *c* as an electron acceptor. Instead, we found that it utilizes molecular oxygen as an acceptor ultimately resulting in the formation of hydrogen peroxide [81]. As plant SOX is localized in the peroxisomes, we suggested that it is involved in a sulfite regulatory pathway in this organelle. Increased levels of sulfite lead to the inhibition of peroxisomal catalases, resulting in an increase in the hydrogen peroxide concentration that subsequently nonenzymatically oxidizes a second molecule of sulfite [81] (Figure 7).

Later, Russ Hille’s group demonstrated that the primary reaction product with oxygen is superoxide that subsequently decays spontaneously to form hydrogen peroxide [80]. Interestingly, vertebrate SOX was initially found not to react with oxygen, which remained enigmatic for the following years. However, Abdel Ali Belaidi, a graduate student from Günter Schwarz’ laboratory, discovered that the removal of the heme domain from mouse or human SOX enables both enzymes to accept oxygen as the electron acceptor, suggesting that the very high rate of electron transfer from Mo to heme prohibits any reaction with molecular oxygen [82]. Another property of plant SOX has to be mentioned here, and this is the extremely high stability of the enzyme. As the biosensor, the enzyme was immobilized into an electrochemical enzyme reactor where it showed superior results in terms of detecting sulfite in food stuff and fruit juices [83]. Along those lines, apo-SOX was also rather stable and used as a novel tool for the detection and quantification of Moco [84].

**Figure 8:**
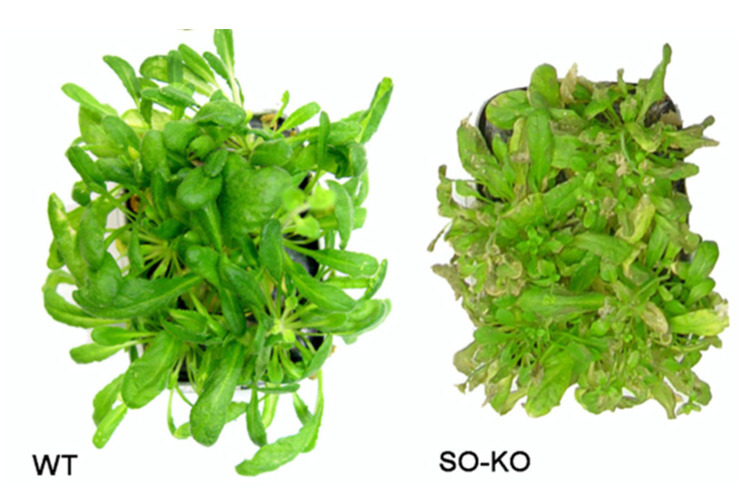
SO_2_ fumigation of both *A. thaliana* SOX knock-out plants (SO-KO) and wild-type (WT) using 50 mM sodium bisulfite for 3 days. Modified after [87].

When SO_2_ is converted into sulfite in an aqueous solution, it is a nucleophilic agent that is able to attack innumerous substrates [85,86] by opening S–S bridges. This reaction—so-called sulfitolysis—causes the inactivation of proteins and, as a consequence, leads to a severe reduction in plant growth or even cell death. What is the physiological role of SOX in plants? Sulfur assimilation (via sulfite and sulfide) proceeds in the chloroplasts and is vital for the survival of the plant cell. As we found At-SOX in peroxisomes rather than in chloroplasts, this suggests that the function of SOX is not related to the chloroplast-based sulfur assimilation pathway. Obviously, the compartmentalization of sulfur assimilation and sulfite oxidation in different organelles allows plants to coregulate these opposing metabolic demands. The knock-out of the gene for At-SOX would give insights into its role in plant metabolism; however, the loss of SOX activity was not lethal, whereas mutations in the vertebrate counterparts are. Arabidopsis plants with an At-SOX knock-out were still alive and looked normal. Only when the plants were exposed to a highly SO_2_-enriched atmosphere did they show a significant decrease in vitality and bleaching under conditions that were phenotypically not harmful for the wildtype control (Figure 8). 

**Figure 9:**
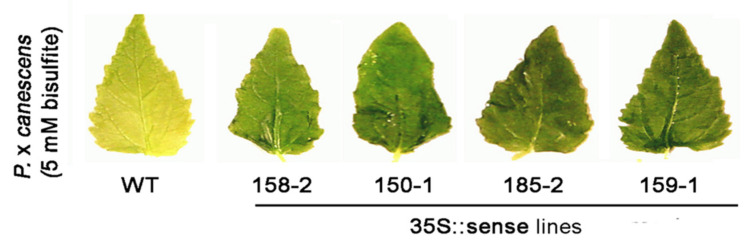
SO_2_ fumigation of poplar (*P.* x *canescens*) lines over-expressing Arabidopsis SOX under control of the 35S promoter with 5 mM sodium bisulfite for 5 days in comparison to the wildtype (WT). Modified after [87].

The additional sulfite was metabolized to cysteine and glutathione as oxidation to sulfate was precluded. In these knock out-mutants, the reductive sulfur assimilation pathway seems to be the only possibility to detoxify excessive sulfite, thus resulting in elevated levels of cysteine and glutathione. At-SOX is highly expressed in the plant (=0.1% of total leaf protein [81]). This observation provides evidence that, under normal conditions, the SOX expression is sufficiently high to ensure the detoxification of increased amounts of sulfite. Only exceedingly high amounts of sulfite as generated by the high-SO_2_ dosages seem to cause an upregulation in the SOX expression. The overexpression of SOX in a poplar model for a SO_2_-sensitive woody plant demonstrated the benefit of elevated SO expression as these plants showed increased tolerance against SO_2_ fumigation (Figure 9). 

When analyzing the transcriptome of At-SOX knock-out plants and comparing it to the wild-type control, we observed remarkable and broad regulative responses at the mRNA level, especially in transcripts related to sulfur metabolism enzymes, as well as in those related to stress response and senescence ([88]). These omics analyses identified two novel candidates for involvement in SO_2_ detoxification beyond SOX: an apoplastic peroxidase (a heme-enzyme) that shows sulfite oxidation activity [89], and defensins as putative cysteine mass storages [88]. 

At last remains the question: Why has SOX been preserved throughout plant evolution? Plants as sessile organisms have to cope with the smoke conditions at a given location. In contrast to the continuous but moderate SO_2_ exposure at dormant volcanos ([90]), plants near wildfires had to react within several hours to counteract high concentrations of toxic gases, among them SO_2_. The first line of defense is a tight control of gas uptake by regulating stomatal aperture, and a second line is the upregulation of sulfur assimilation like cysteine production in the chloroplasts and the back-oxidation of sulfite to sulfate in the peroxisomes leading to the storage of sulfate in the vacuole. This involves SOX. We analyzed trees near recent wildfires and found that beech trees use an efficient up-regulation of SOX activity while oak trees hold a constitutively high SOX pool available which is 10-fold higher than in beeches ([91]). In summary, the activity of plant SOX ensures that the intracellular levels of the otherwise deleterious sulfite ion remain at acceptably low levels. 

## 4. Human SOX-Deficiency and Therapy—A Personal View by Günter Schwarz (Years 2002 Onward) 

The first patient with a defect in SOX was reported in *Science* back in 1967 by S. Harvey Mudd [20], a trained medical doctor who stayed in biochemical research throughout his life and became a world-leading figure in the field of inborn errors in methionine metabolism, with a focus on remethylation disorders [92]. Besides SOX deficiency, Mudd discovered numerous disorders in the methylation and remethylation cycle of methionine as well as the reverse trans-sulfuration pathway. 

In the following years, initial insights regarding the underlying cause of neurodegeneration were collected, such as the potential excitotoxic action of S-sulfocysteine, a metabolite strongly accumulating in SOX deficiency [93]. As introduced in Section 1, Jean Johnson and K.V. Rajagopalan were pioneers in the molecular analysis of SOX deficiency. Together with Vivian Shih, in 1977 a second patient with SOX deficiency was reported at Harvard Medical School (USA) [26]. A few years later, Jean Johnson could further dissect the molecular basis of SOX deficiency in the second patient, and it turned out that this patient was lacking Moco [94] thus being the first documented Moco deficiency patient. More than three decades later, we found the explanation for the absence of the SOX apoprotein to be due to a maturation defect of SOX in the mitochondrial intermembrane space in the absence of Moco [52]. Vivian Shih’s index patient survived until their mid-forties (F. Eichler, Mass. General Hospital, personal communication). Today, it is commonly accepted that both SOX- and Moco-deficient patients show a remarkable overlap in clinical presentation, underlining the key importance of Moco for SOX function. In contrast to SOX patients, patients suffering from Moco deficiency also present altered biomarker profiles for purine metabolites (accumulation of hypoxanthine/xanthine, lack or strong reduction in uric acid).

From 1980 to 2000, Jean Johnson was the leading figure in deepening our understanding of SOX and Moco deficiency. She identified urothione as the catabolic end-product of Moco [95], and reported together with Vivian Shih two genetic complementation groups for human Moco deficiency patients [96], and found a first diffusible precursor of Moco [97], which became later instrumental for the development of the first treatment of Moco deficiency [98]. Based on those studies, Moco deficiency (MoCD) classified type A and type B patients, and later an ultra-rare and even more severely affected type C category completed the picture. The latter aspects of the genetics of Moco deficiency are linked to Jochen Reiss, a human geneticist from Göttingen University (Germany), who, in collaboration with our Braunschweig lab, discovered *MOCS1* as Moco synthesis gene [99], while *MOCS2*, *MOCS3* and *GPHN* (Gephyrin) were identified and characterized in Braunschweig [100,101]. We tried to name the human genes “mcs”, for “Moco synthesis”, but this gene symbol was already assigned for a muscle gene, so we chose “mocs”. 

Starting out in plant biochemistry, I was fascinated by the level of functional, genetic, and structural conservation of Moco synthesis and the SOX family of enzymes. Given the unprecedented finding of the *gpnh* codes for a moonlighting protein (Gephyrin) in Moco synthesis and receptor clustering, my lab became interested in studying Gephyrin function and contributing to understandings of its structure [102,103], synaptic function [84,104,105], and role in different forms of disease [106,107].

The aspect of a neurodegenerative disorder in Moco and SOX deficiency also fascinated me early on and was further enhanced by a conference on pterins and folates where I met the synthetic chemist Berthold Fischer. Triggered by an incorrectly depicted chemical structure of Precursor Z, the first intermediate of Moco synthesis, we started a collaboration expressing and purifying this unknown pterin compound from *E. coli* cells. Berthold Fischer recruited a chemistry student from Spain to work with him at Bochum University (Germany); however, shortly after he started, Berthold had to switch labs and moved to Singapore. He asked me if I would be willing to continue the project with his student Jose Angel Santamaria-Araujo, and of course I agreed and supervised Jose’s Ph.D. project in my remaining years in Braunschweig. After graduation, Jose joined my new group at Cologne University (Germany), and ever since, we have been working together as colleagues, partners, and friends.

Jose Santamaria uncovered the chemical structure of Precursor Z [108], and as a result of those findings, we named the molecule cyclic pyranopterin monophosphate (cPMP). Together with Jochen Reiss, we established a successful replacement therapy of Moco deficiency in a mouse model he developed [98,109]. These studies were some of the very few experiments we had conducted in the last 25 years that showed unequivocally clear results with the very first “shot”. Mocs1-deficient mice that received regular cPMP treatment by intrahepatic injections showed normal survival and development, while untreated animals or animals in which treatment was discontinued died within 7–10 days [98]. Based on those studies, we applied for funding by the Federal Ministry of Education and Research supporting applied projects in the Lower Saxony State. It happened that 36 h before closing of the final call of the program, Ralf Mendel and I learned that such a funding opportunity exists, and it needs no more words to explain how much energy this opportunity released in drafting the proposal over-night. At that time, the review panel was very skeptical that researchers from a Plant Biology Department may manage to develop a drug that could be potentially used in human patients. Nevertheless, we received the very last funding of nearly EUR 1 million for three years and developed a biotechnological and upscaled process for purifying cPMP from recombinant *E. coli* cells.

In 2008, a few months before finishing our cPMP-production project, we were contacted by clinicians from Monash University Children’s Hospital in Melbourne (Australia), asking whether we could help with the diagnosis and potential treatment of a patient with Moco deficiency. In an unprecedented and highly collaborative effort supported by clinicians, parents and family members, clinical scientists, and legal staff, the first human treatment of Moco deficiency with bacteria-derived cPMP was started on 8 June 2008 [110] by Alex Veldman in Melbourne. This milestone was reached in less than two weeks and required the shipment of all cPMP we had in stock in Cologne down to Melbourne for chemical analysis and certification, the investigation of the blood and urine of the patient allowing for the diagnosis of MoCD type A, and most importantly, drafting a treatment plan including dose-finding. Two days before treatment was initiated, the legal counsel of Monash University (Sydney) requested a formal approval of this highly experimental treatment by the Supreme Court of Australia in Sidney, which was approved on a Friday afternoon. The entire ‘story’ became public following an accidental notification by a journalist who found the records of the supreme court being released upon a one-year embargo, triggering a press release by Monash University followed by the story of Baby Z going around the globe, time zone by time zone. 

Today, the treatment of MoCD type A with cPMP (now named Fosdenopterin by Origin Biosciences Inc. (Boston, MA, USA) and marketed by Sentynl Therapeutics Inc. [Solana Beach, CA, USA]) is a therapy approved by the FDA (2021) and EMA (2022). However, for Baby Z, the first patient showing immediate and sustained biochemical response [110], pre-existing brain damage could not be completely resolved, resulting in severe neurodegeneration with cerebral palsy. In the following years, more patients received cPMP treatment following a compassionate use/patient-named treatment access [111]. Patients that received treatment early after birth showed still showed normal development, demonstrating the effectiveness of the substitution approach. Therefore, in the future, it is most important that early diagnosis is made to prevent brain damage and that treatment is provided to as many patients suffering of MoCD type A as possible.

Despite the progress in treating MoCD type A patients representing the largest fraction of Moco-deficient patients suffering of SOX deficiency, we still need to find therapies for MoCD type B and SOX deficiency patients. In a recent review, we proposed a number of interventions that are based on a better understanding of the underlying disease mechanism [112]. While, in the past, dietary restrictions only showed positive results in a few late-state and milder forms of SOX deficiency [113], this approach needs a more systematic evaluation using an inducible/conditional mouse model. Blocking the excitotoxic action of SSC in the brain by inhibiting NMDA receptor signaling using approved drugs such as memantine may suppress or delay rapidly progressing neurodegeneration. Most promising are strategies that would target sulfite scavenging using either small molecules or enzyme-based clearance. Here, any small molecule oxidized thiol could serve as sulfite target. Two aspects will be important for such an approach, including the low toxicity of the resulting S-sulfoxidated species as well as high renal clearance. For example, cysteamine, a drug used to treat cystinosis, could serve such a sulfite-scavenging function. Also, enzyme substitution either by applying a modified form of SOX or mRNA therapy are promising new approaches. A recent study in Moco-deficient *Caenorhabditis elegans* demonstrated that the lethal symptoms of MoCD in worms can be suppressed by feeding the worm Moco-containing *E. coli* or providing an in vitro source of protein-bound Moco [114,115], thus protecting the worm from sulfite toxicity. This finding suggests that not only cPMP but also Moco may be used to treat MoCD in the future. The molecular mechanisms used by the worm to retrieve Moco from its diet are presently under investigation.
